# Artificial intelligence-based diagnosis of asbestosis: analysis of a database with applicants for asbestosis state aid

**DOI:** 10.1007/s00330-022-09304-2

**Published:** 2022-12-26

**Authors:** Kevin B. W. Groot Lipman, Cornedine J. de Gooijer, Thierry N. Boellaard, Ferdi van der Heijden, Regina G. H. Beets-Tan, Zuhir Bodalal, Stefano Trebeschi, Jacobus A. Burgers

**Affiliations:** 1 Department of Radiology, The Netherlands Cancer Institute, Amsterdam, The Netherlands; 2 Department of Thoracic Oncology, Netherlands Cancer Institute, Amsterdam, The Netherlands; 3 Technical Medicine, University of Twente, Enschede, The Netherlands; 4 GROW School for Oncology and Developmental Biology, Maastricht University Medical Center, Maastricht, The Netherlands; 5 Department of Robotics and Mechatronics, University of Twente, Enschede, The Netherlands; 6 Institute of Regional Health Research, University of Southern Denmark, Odense, Denmark

**Keywords:** Asbestos, Asbestosis, Tomography, X-ray computed, Respiratory function tests, Artificial intelligence

## Abstract

**Objectives:**

In many countries, workers who developed asbestosis due to their occupation are eligible for government support. Based on the results of clinical examination, a team of pulmonologists determine the eligibility of patients to these programs. In this Dutch cohort study, we aim to demonstrate the potential role of an artificial intelligence (AI)-based system for automated, standardized, and cost-effective evaluation of applications for asbestosis patients.

**Methods:**

A dataset of *n* = 523 suspected asbestosis cases/applications from across the Netherlands was retrospectively collected. Each case/application was reviewed, and based on the criteria, a panel of three pulmonologists would determine eligibility for government support. An AI system is proposed, which uses thoracic CT images as input, and predicts the assessment of the clinical panel. Alongside imaging, we evaluated the added value of lung function parameters.

**Results:**

The proposed AI algorithm reached an AUC of 0.87 (*p* < 0.001) in the prediction of accepted versus rejected applications. Diffusion capacity (DLCO) also showed comparable predictive value (AUC = 0.85, *p* < 0.001), with little correlation between the two parameters (*r*-squared = 0.22, *p* < 0.001). The combination of the imaging AI score and DLCO achieved superior performance (AUC = 0.95, *p* < 0.001). Interobserver variability between pulmonologists on the panel was estimated at *alpha* = 0.65 (Krippendorff’s alpha).

**Conclusion:**

We developed an AI system to support the clinical decision-making process for the application to the government support for asbestosis. A multicenter prospective validation study is currently ongoing to examine the added value and reliability of this system alongside the clinic panel.

**Key Points:**

*• Artificial intelligence can detect imaging patterns of asbestosis in CT scans in a cohort of patients applying for state aid.*

*• Combining the AI prediction with the diffusing lung function parameter reaches the highest diagnostic performance.*

*• Specific cases with fibrosis but no asbestosis were correctly classified, suggesting robustness of the AI system, which is currently under prospective validation.*

**Supplementary Information:**

The online version contains supplementary material available at 10.1007/s00330-022-09304-2.

## Introduction

Asbestosis is diffuse pulmonary fibrosis emerging after prolonged, mainly occupational, exposure to asbestos [1]. Many countries have banned asbestos in construction and manufacturing [2]. However, due to the long incubation time, many (former) exposed workers now present with asbestosis [3].

Asbestosis patients with occupational asbestos exposure might be eligible for financial compensation [4]. The criteria for obtaining it vary by country, although international attempts have been undertaken for standardization [4–6]. Standardization is hard to achieve. Disagreement among experts [7, 8] on the Helsinki criteria [6] for asbestosis hindered this process. In the Netherlands, the following criteria are legally set for financial reimbursement: 1) computed tomography (CT) imaging, preferably high-resolution CT (HRCT) with fibrosis covering > 5% of the lung area, 2) lung function loss should be present, and 3) occupational asbestos exposure of at least five fiber years (product of the intensity of asbestos exposure times the occupational years [9, 10]). Three independent and experienced pulmonologists review the clinical case and state whether the most likely diagnosis is asbestosis. The majority of votes set the diagnosis for reimbursement. Similar procedures are followed in other countries [11–13].

This law-driven diagnosis does not coincide entirely with the clinical, multidisciplinary board meeting-driven diagnosis. Additionally, a shared limitation is the unknown inter-rater variability, leaving the quality and reproducibility of the final verdict unknown. This could lead to the same patient receiving different diagnoses for unclear cases. Alternatively, obvious cases are still processed by three experts, where their effort could have had more impact analyzing the unclear cases.

We hypothesize that a system based on artificial intelligence (AI) could replicate the assessments of the three experts. AI is a method to automatically extract data patterns from raw data (e.g., CT scans) to predict outcomes of interest (e.g., decision of the pulmonologists’ panel). In this study, we aim to develop and test an AI system to assess applications of subjects with recorded exposure to asbestos, and determine whether they are eligible for financial support. If the AI is certain about its prediction, one pulmonologist could be sufficient to verify the AI assessment. More pulmonologists can be assigned to process the unclear case when the AI is uncertain. The resulting AI algorithm evaluates eligibility and can be implemented uniformly in multiple centers, allowing for increased consistency in handling financial support requests.

## Material and methods

### Datasets

We performed a retrospective analysis on a dataset of prospectively included applicants for financial support, collected by the Dutch Institute of Asbestos Victims (IAS) and the Netherlands Cancer Institute (NKI; Amsterdam/NL) between 05/2014 and 11/2019 [4]. Applicants gave informed consent for the use of their data. CT scans with 3-mm slice thickness/increment were preferred over 1 mm due to hardware constraints. While ≤ 1-mm slices are preferred in clinics for diagnosing ILDs, the GPU cannot process hundreds of ≤ 1-mm slices as one volume.

Exclusion criteria for the current analysis were as follows: no chest-CT scan available, lungs not fully present in the scan, slice thickness > 5 mm, or absence of panel verdict. The dataset was divided between training, validation, and test sets based on a random, reproducible split. Each set consisted of an equal ratio of positive/negative cases. For evaluation, four contradictory cases (interstitial lung disease (ILD) but no asbestosis, or asbestosis but little to no ILD) were held out.

When available, the following lung function tests were retrieved: vital capacity (VC, cm^3^), forced vital capacity (FVC, cm^3^), and diffusing capacity of the lung for carbon monoxide (DLCO, mL/min/mmHg). To compensate for differences in body type, the lung function parameters are denoted in percentage (%) of expected value. To quantify the loss of lung function, Hagmolen Of Ten Have et al adapted the American Medical Association (AMA) classes described by Rondinelli et al [14]. The worst-recorded parameter between FVC and DLCO was converted to an impairment class (AMA class, Table 1). AMA ≥ 2 is regarded as sufficient for financial support (see Supplementary information).

**Table 1:** Table for converting loss of lung function to a specific AMA class (*Guides to the Evaluation of Permanent Impairment* Sixth Edition) FVC and DLCO values are the corrected percentages for age, length, and sex of the predicted normal value

Class	0	1	2	3	4
FVC	≥ 80%	70–79%	60–69%	50–59%	< 50%
DLCO	≥ 75%	65–74%	55–64%	45–54%	< 45%

The parameter with the highest loss determined the AMA class, which in turn was correlated to the extent of the financial reimbursement

### Design of the artificial intelligence

**Fig. 1:**
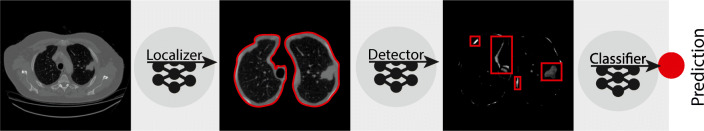
An overview of the AI system with the Localizer, Detector, and Classifier modules. The red outline indicates the areas of interest for each module

We designed an AI system for the assessment of eligibility of asbestosis financial support applications following subsequent steps: 1) identification of the lungs and surrounding tissue in chest-CT scans through a localizer, 2) detection of anomalies within the lungs with a detector, and 3) automatic assessment of eligibility through a classifier, based on the CT scan and the anomalies found in 2). These modules function synchronously within the overall AI diagnostic system (Fig. 1). The code is publicly available on the AI repository of our department^1^, enabling other researchers to redo a similar study.

#### Localizer

This module aims to detect and segment the lungs. We reused an AI model^2^ of LaLonde et al [15]. Once it identified the lungs, we automatically removed all non-lung pixels from the image. This facilitates the subsequent analysis, ensuring that they will only be performed on lung tissue.

#### Detector

The goal of this module is to highlight anomalies in the lungs. We based the design on a set of algorithms for anomaly detection, called variational autoencoders (VAE) [17]. In our case, we trained a VAE on a dataset of chest-CTs (see Supplementary information). By training this network on healthy CT slices, the VAE learns to synthesize healthy lung structures. When a CT with lung anomalies is presented to the network, the VAE will reconstruct those abnormal regions of the lungs poorly, since they are not learned during training. This phenomenon allows us to highlight the anomalies, effectively creating an anomaly heatmap (details in Supplementary information).

#### Classifier

This module aims to identify patients who received a positive assessment for asbestosis financial support. We based our design on the ResNet architecture [18], which is commonly employed for image classification tasks. We trained the network using the CT+anomaly heatmap as input and the asbestosis panel verdict as training objective, where the cross-entropy loss function quantified the difference between AI prediction and panel verdict. Once trained, the network made predictions between 0 and 1, to be interpreted as a probability, with 0 being no evidence to support positive assessment and 1 being the opposite.

### Data curation and labels

To minimize differences between imaging protocols and artifacts from foreign metal bodies, e.g., pacemakers, all Hounsfield units (HU) were clipped between −1024 HU (air) and 3072 HU (dense bone) [19] and scaled on 0–1 interval. To include adjacent tissue of the thoracic wall (such as pleural plaques/thickening), we dilated the segmentations through morphological operators with a kernel of 13 × 13 × 5 voxels. Subsequently, they were visually inspected and adjusted in 3DSlicer (v4.10) [16] if the pleura was not present in the dilation.

The segmented lungs in the CT were cropped to 192 × 192 × 96 (sagittal, coronal, axial) and rescaled where needed due to hardware constraints. The ground truth, i.e., the *label*, was implemented in two configurations: hard and soft. The hard labels were binary (i.e., asbestosis or not), whereas the soft labels reflected the panel’s agreement (i.e., ratio of positive assessments). These soft labels were implemented to investigate whether the AI could replicate that level of agreement. When pulmonologists disagree, soft labels penalize uncertain AI predictions less than hard labels. Specifically, an AI prediction during training of 0.5 (uncertain) is closer to the fraction of pulmonologists positive: 0.33 (1/3 pulmonologist, 0.17 off target, soft label) than the final verdict of the panel: 0 (1/3 pulmonologist, 0.5 off target, hard label).

### Statistical analysis

To evaluate the panel’s inter-observer variability, we calculated Krippendorff’s alpha, where *ɑ* = 1 reflects perfect agreement and *ɑ* = 0 disagreement [20]. The performance of the models was evaluated using the ROC-AUC and standard measures of accuracy, sensitivity, specificity, and positive and negative predictive values. We performed McNemar’s test to test for significant differences in performance between different methods. The correlation between lung functions and AI predictions was estimated via r-squared (*r*^2^). To visualize the areas where the model focused on in the CT scan, we traced the activations back to the input, creating so-called saliency maps [21]. These saliency maps can be interpreted as overlays, which contain higher values on areas of the CT that contribute more towards the final prediction. Furthermore, we aimed to develop models that did not produce significant outliers, i.e., incorrect predictions close to 0 and 1. This improves the explainability of predictions to both applicants and physicians.

## Results

### Study cohort

In total, we retrospectively collected *n* = 523 applications for financial support. Median age was 75 years (IQR 69–80). The dataset contained two female applicants (0.4%). The pool of pulmonologists consisted of *n* = 23 experts, with 20 years of experience at the median (CI: 16–27). For each application, *n* = 3 pulmonologists were assigned to process the application, with each pulmonologist having the same probability of getting assigned.

At the database lock of November 2019, *n* = 16 did not receive an assessment of the panel. Of the *n* = 507 remaining cases, *n* = 233 applicants received a positive assessment (46.0%), with *n* = 166 (71.2%) unanimously. The remaining *n* = 274 applications did not meet the criteria, with *n* = 219 (79.9%) unanimous assessments. Inter-observer variability between pulmonologists was estimated at *alpha=*0.65 (Krippendorff’s alpha), with 75.9% unanimously (*n* = 385).

For AI development, *n* = 88 additional applications were excluded: *n* = 78 for absence of fully imaged lungs in the CT and *n* = 10 for CT slice thickness > 5 mm. A total of *n* = 419 formed the study dataset. The excluded cases did not differ significantly by age or lung function. CT scans protocol were heterogeneous due to the multicenter origin of the data (median, CI): voltage (120 kVp, 118.7–121.3), tube current (194 mA, 177.4–210.6), slice thickness (3 mm, 2.85–3.14).

### AI training

We split the dataset into a training (*n* = 263), validation (*n* = 64), and test set (*n* = 88), based on a train-test split of 80/20 [22], with a reproducible pseudo-randomization (sklearn v0.24.1). We ran experiments with different label formats (i.e., *soft,* which reflects the agreement, and *hard*, which is binary), and with and without anomaly heatmap.

### AI predictive performance

Soft labels combined with the anomaly heatmap yielded the best performance in all metrics (Table 2). Soft labels yielded a more uniform prediction distribution between 0 and 1 compared to hard labels (soft std = 0.33, hard std = 0.40, *p* < 0.001). Following the McNemar test comparing the predictions, the soft label model yields higher performance (AUC = 0.87, CI: 0.78–0.94, *p* < 0.001) than the hard label model (*p* = 0.017) Moreover, soft labels with anomaly heatmap performed significantly better than soft labels without heatmap (*p* = 0.042), indicating that both the soft labels and anomaly heatmap were required for increased performance. While the setup without anomaly heatmap and with hard labels scored best on sensitivity and negative predictive value, the overall performance of the soft label with anomaly heatmap was significantly better as well (*p* = 0.017, McNemar test) (Table 2). The model yields an accuracy of 0.82 (0.74–0.90), with a sensitivity of 0.76 (0.62–0.88), and a specificity of 0.87 (0.77–0.96). Positive and negative predictive values were 0.84 (0.71–0.95) and 0.81 (0.69–0.91), respectively.

**Table 2:** The results of the different tested setups of AI models

Label	Anomaly heatmap	ACC	SENS	SPEC	PPV	NPV	*p* value
Hard	No	0.65	**0.93**	0.40	0.58	**0.86**	0.017
Soft	No	0.66	0.78	0.55	0.60	0.74	0.042
Hard	Yes	0.65	0.46	0.81	0.68	0.63	0.043
Soft	Yes	**0.82**	0.76	**0.87**	**0.84**	0.80	**-**

The bold number shows the maximal performance in terms of the metric of that column. Hard labels are binary, while soft labels reflect the agreement of the pulmonologists on the panel. The *p* values were calculated with the McNemar test compared to the best-performing model (soft labels with anomaly heatmap)

**Fig. 2:**
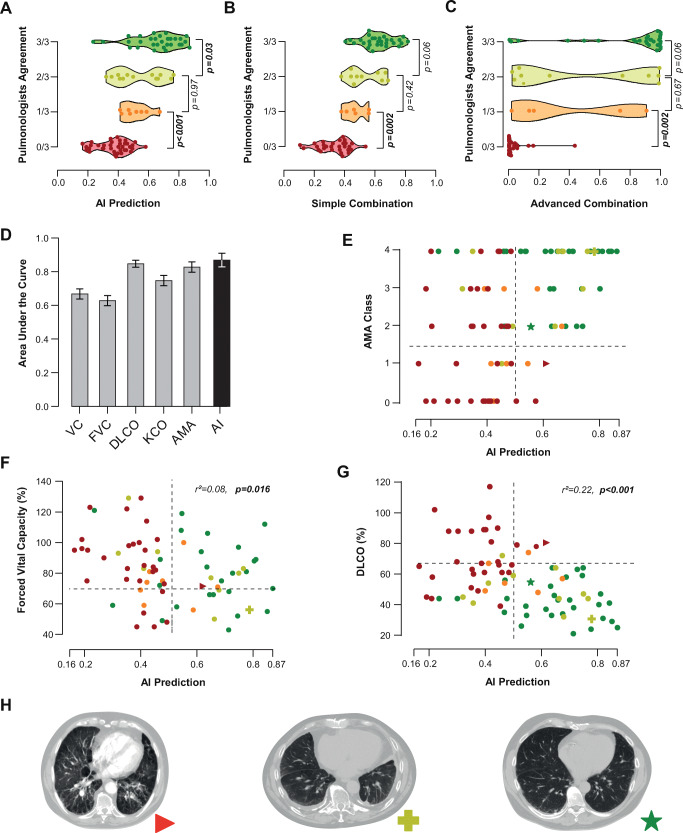
The colors reflect the agreement of the panel of pulmonologists: asbestosis negative (red dots), one out of three positive (orange), two out of three positive (light green), asbestosis positive (green dots). **A**–**C** Violin plots on different setups of prediction. The y-axis shows the agreement of the panel of pulmonologists. The x-axis shows the predicted probability of asbestosis. *p* < 0.001 between the predictions in classes 0 and 3 for all setups. **A** The prediction of the AI model. **B** The score of the AI model linearly weighted with the DLCO. **C** The prediction of the AI model that took both the CT and the DLCO as input. **D** Bar plot of the diagnostic value (expressed as AUC) of the different lung function parameters, the AMA class, and the AI model. **E**–**G** Probability of asbestosis predicted by the AI model versus (**E**) AMA, (**F**) FVC, and (**G**) DLCO. The horizontal dotted line indicates the cutoff value for lung function loss; the vertical dotted line indicates the cutoff of the AI prediction. **H** Several cases where the amount of fibrotic tissue does not reflect the diagnosis of the pulmonologists. The symbols of each example are visualized in **E**–**G** when the respective lung function parameter of the patient is known

The distribution of the predicted scores is shown in Fig. 2A, stratified according to the agreement of pulmonologists in the panel (i.e., number of pulmonologists that gave a positive assessment). We performed visual analysis of outliers where all three pulmonologists were positive, but the model prediction was negative (*n* = 5). Most of those applicants (*n* = 4) had a severe reduction in lung function, but the fibrosis in the CT scans did not reflect this.

### Integration of lung function tests

DLCO yielded predictive performance close to the AI model (AUC = 0.85, CI: 0.80–0.89, *p* < 0.001). The remaining lung function parameters yielded lower results: AUC = 0.67 for VC (CI: 0.60–0.72, *p* < 0.001), AUC = 0.63 for FVC (CI: 0.56–0.68, *p* < 0.001), and AUC = 0.83 for AMA (CI: 0.78–0.87, *p* < 0.001).

Interestingly, the DLCO showed a weak correlation with the AI prediction (*r*^2^ = 0.22, *p* < 0.001), suggesting they can be independent predictors of asbestosis. We tested a simple combination, formalized as the average between AI score and DLCO value — ((1 − *DLCO*) + *AI*)/2; and an advanced combination strategy, where the DLCO is added as additional input to the AI system.

The simple combination yielded an AUC of 0.95 (0.89–0.98, *p* < 0.001), with an accuracy of 0.84 (0.76–0.92), a sensitivity of 0.77 (0.63–0.89), and a specificity of 0.91 (0.81–1.00). Positive and negative predictive values were 0.91 (0.80–1.00) and 0.78 (0.65–0.90), respectively. Furthermore, from the distribution of the scores, this setup reported no false negative or false positive under 0.35 and above 0.60, respectively (Fig. 2B).

The advanced combination strategy yielded an AUC of 0.92 (0.86–0.97, *p* < 0.001), an accuracy of 0.84 (0.76–0.92), with a sensitivity of 0.74 (0.60–0.87), and a specificity of 0.94 (0.85–1.0). Positive and negative predictive values were 0.94 (0.83–1.0) and 0.77 (0.64–0.89), respectively. The spread of predictions in agreement with the pulmonologists was wider, as shown in Fig. 2C. More specifically, this model predicts more CT scans closer to either 0 or 1 than the AI and the simple combination do.

Following the AUC (Fig. 2D) and outliers (interpretability), the simple combination of AI+DLCO was considered the best model. Compared to the advanced combination, it yielded a distribution of predictions with lower standard deviation and was more interpretable due to DLCO weighting apart from the AI model.

### AMA class decomposition

To meet the requirement of lung function loss for financial reimbursement, AMA ≥ 2 is needed. Figure 2E shows how the AI prediction distributes over the AMA classes. When decomposing the AMA class in FVC and DLCO, the differences in predictive values become noticeable. FVC values (Fig. 2F, AUC = 0.63) were more scattered than the DLCO values (Fig. 2G, AUC = 0.87). The held out cases with FVC or DLCO reported (Fig. 2H) show how the specific cases interact with the AI prediction and the lung function parameters in Fig. 2E–G.

### Visual interpretation

**Fig. 3:**
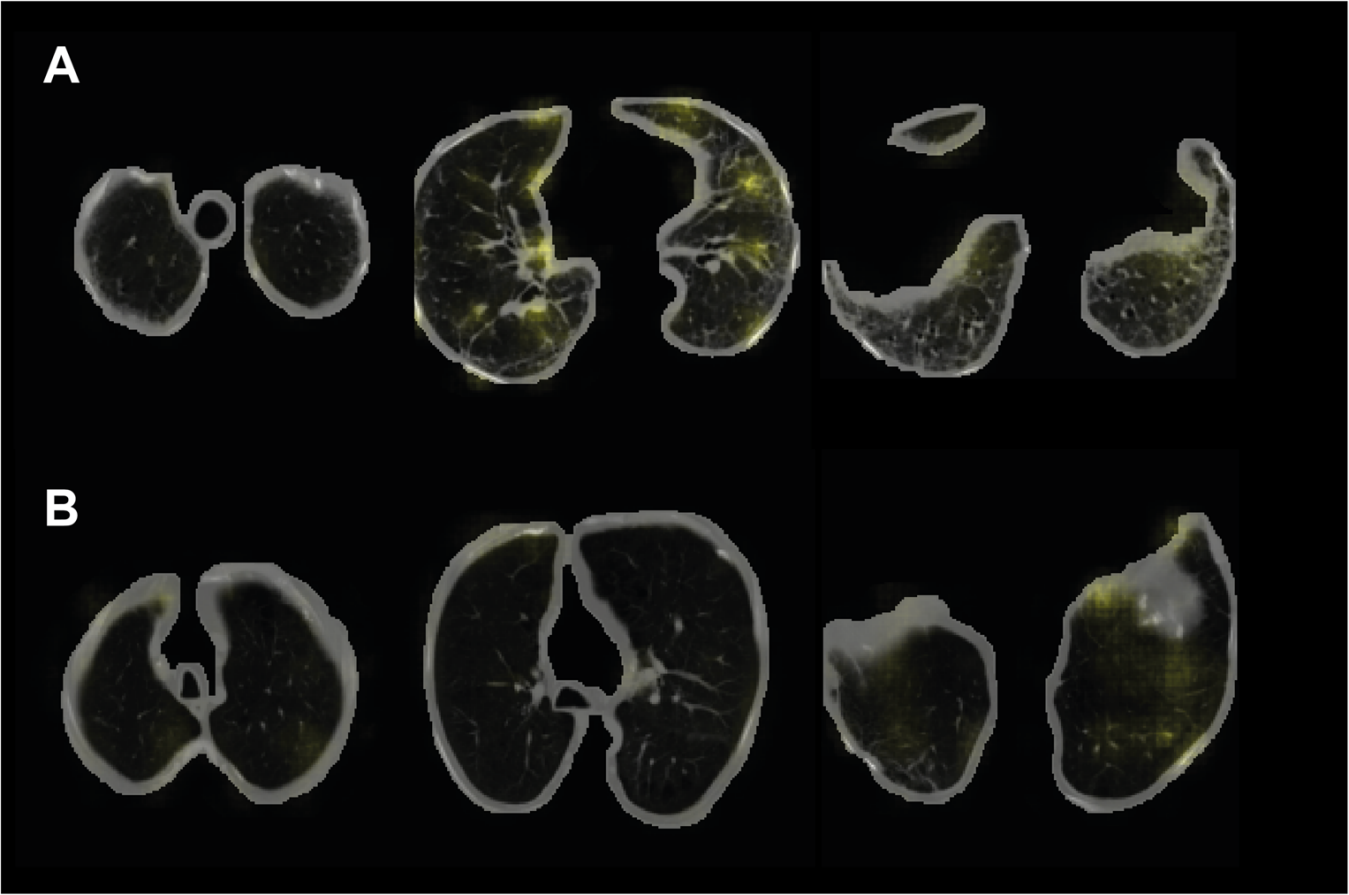
Saliency map yielded by the AI model of two CT scans in the test set. The areas in yellow represent the attention of the model. The left side shows a slice from the top of the lungs, the middle a slice in the middle of the lungs, and the right side a slice from the bottom of the lungs. **A** CT scan where 3/3 pulmonologists were positive and the model yielded a high probability of asbestosis (0.81). **B** CT scan where 0/3 pulmonologists were positive and the model yielded a low probability of asbestosis (0.19)

To enhance interpretability, we generated saliency maps showing that the asbestosis-positive cases show more activations than the asbestosis-negative cases (Fig. 3). From visual inspection, we can see that the CT scan with visible ILD yields more activations, indicating that the AI system learned to identify ILD. In the CT scan where ILD is barely visible, there were hardly activations of the AI system.

## Discussion

The current process for the assessment to determine the eligibility for financial support of workers who had been in contact with asbestos is laborious and costly, and has high intra-observer variability [4]. This study aimed to automate and standardize this process via artificial intelligence (AI) [23]. To do this, we have implemented an AI system [18] that uses thoracic CT scans to replicate the assessment of a panel of three pulmonologists (as required by national law). Our AI model to automatically classify the eligibility of applicants for state aid for people with asbestosis yielded significant results and classified eligible applications with high accuracy. The best-performing lung function parameter DLCO showed comparable results [24]. The combination of the AI+DLCO yielded a superior predictive performance than either AI or DLCO alone.

By accounting for the uncertainty in the pulmonologists’ assessment (i.e., soft labels), our model reached higher accuracy than the same model that ignores it (i.e., hard labels). We hypothesize that the soft labels enable the AI to learn the uncertainty in specific cases, while the hard labels promote predicting either 0 or 100% probability. This is supported by the difference in standard deviation in the predictions of the soft/hard label AI models. The level of agreement observed in the panel of pulmonologists is lower than the cutoff considered sufficient for reliable results [20]. AI systems are notorious for their susceptibility to uncertainty in the provided labels [25]. The implementation of soft labels is based on the assumption that the levels of agreement between pulmonologists reflect a true, underlying level of uncertainty, which is also present in multidisciplinary meetings of interstitial expert teams [26]. The ability of the model to replicate the uncertainty suggested that it is not random but rather dependent on clinical or biological characteristics. Pure binary models would allow only for two outcomes: accept or reject. Due to the ability to replicate the uncertainty, we accepted a third outcome: process further. We envision the unsure cases (AI probability between 0.35 and 0.6) getting extra attention from the panel, while one pulmonologist handles the clear cases (< 0.35, > 0.6). Therefore, there will always be a need for a (multidisciplinary) panel.

Lung function tests played a significant role in identifying false-negative cases where all pulmonologists returned a positive assessment, and the AI model returned a negative one (Fig. 2A, B: difference in top rows). This suggested that the lung function tests largely drove the verdict for these applicants. In other words, the AI model could not detect a loss of lung function based on the CT scan of these applicants. This was supported by the weak–moderate correlation observed between DLCO and AI model.

DLCO contributed to the diagnostic accuracy of our model, whereas the inclusion of FVC only deteriorated the ability to distinguish between positive and negative applications. Two reasons might explain this phenomenon: [1] the pulmonologists (unconsciously) based their verdict mainly on DLCO, while not taking FVC into account, and [2] decreased DLCO correlates with diffuse fibrosis, where the pulmonologists based their verdict mainly on the radiologic features of diffuse fibrosis. These findings align with Nogueira et al, who found that DLCO correlated most to the short-term progression of abnormalities in HRCT [24]. It may be beneficial for the panel to make DLCO measurements obligatory for more consistent, standardized, and objective evaluations.

Although AI models contain biases on their own [28], they could help overcome human bias [27] and ensure a fairer public health policy in this situation. Our work aligns with current literature that suggests automatic AI systems for ILD classification could improve patient healthcare [29].

Our study contained several limitations. Because of missing lung function parameters, each lung function performance was computed on slightly different sub-cohorts. Due to hardware limitations in the operational resolution of the AI algorithm, we had to downsample the CT images, blurring finer-grained structures like fibrosis [30]. While the AI model performs excellently in classifying cases where the panel is anonymous, it lacks explainability. Saliency maps indicate where the AI model is “looking at” but are insufficient in explaining why a patient’s application got accepted/rejected. There will always be a need for humans in these processes. Another improvement would be to include the cumulative asbestos exposure as input. Furthermore, our analysis was only retrospectively validated. Most AI algorithms are not validated prospectively [31], and the value of commercially available products is often not substantiated by peer-reviewed publications [32]. Therefore, we chose to validate our simple combination of the AI model and the DLCO in a prospective setting (PROSBEST, Trial NL9064).

Given these results, we can envision an automatic and standardized diagnostic AI system of the application based on the CT scan and lung function tests [33]. Further research in other clinical settings should reveal whether the method used might be useful in diagnostication of patients with interstitial lung disease in general.

## Conclusion

We developed an AI model to diagnose asbestosis in applicants for financial reimbursement according to parameters set by Dutch law. Classification models based on only the CT scan and a combination of the CT scan and the lung function test were quantitatively and qualitatively assessed. The model based on the CT scan and the DLCO was superior to the other models and reached excellent diagnostic accuracy. Whether this method could be implemented in other diagnostic settings for asbestosis or interstitial lung diseases is under investigation.

## Supplementary Information

Figure S1 The architecture of the implemented 3D ResNet. The left column shows the encoder, where the image is downsampled through subsequent ResNet blocks to generate a prediction. The right column shows the ResNet block architecture. The black arrows represent the connections of the blocks. The blue arrows represent the identity connections, where the output of an activation layer is added to the input of another convolutional layer. (DOCX 23.4 kb)

## Abbreviations

AI: Artificial intelligence
AMA: American Medical Association
AUC: Area under the curve
CI: Confidence interval (95%)
CT: Computed tomography
DLCO: Diffusing lung capacity for carbon monoxide
FVC: Forced vital capacity
GPU: Graphics processing unit
HRCT: High-resolution computed tomography
HU: Hounsfield units
IAS: Dutch Institute for Asbestos Victims
NKI: Netherlands Cancer Institute
ROC: Receiver operating characteristic
VAE: Variational autoencoder
VC: Vital capacity
VO2: Maximum oxygen uptake

## Footnotes

1 https://github.com/nki-radiology/asbestosis

2 https://github.com/lalonderodney/SegCaps

## Declarations

### Guarantor

The scientific guarantor of this publication is Kevin Groot Lipman.

### Statistics and biometry

No complex statistical methods were necessary for this paper.

### Methodology

• retrospective

• diagnostic or prognostic

• performed at one institution
