# Supplementary material for: Artificial intelligence-based diagnosis of asbestosis: analysis of a database with applicants for asbestosis state aid
Source: Eur Radiol. 2022 Dec 26;33(5):3557–65. doi: 10.1007/s00330-022-09304-2 (PMC10121486; doi:10.1007/s00330-022-09304-2)
Supplement: Supplementary file 1 — The architecture of the implemented 3D ResNet. The left column shows the encoder, where the image is downsampled through subsequent ResNet blocks to generate a prediction. The right column shows the ResNet block architecture. The black arrows represent the connections of the blocks. The blue arrows represent the identity connections, where the output of an activation layer is added to the input of another convolutional layer. (DOCX 23.4 kb) [file 330_2022_9304_MOESM1_ESM.docx]

**Supplementary Materials**
*Application Procedure*
Currently, the members of the committee give their approval for a positive asbestosis diagnosis if three criteria are met: (1) the patient has a sufficient history of occupational asbestos exposure, (2) the surface of the lung parenchyma in the CT scan of the patient is at least 5% covered with fibrosis, and (3) the patient has a reduced lung function. This is the legal diagnosis for asbestosis, rather than the clinical one. A committee of three pulmonologists evaluates whether the applicant fulfills the three criteria for a positive assessment. They are blinded to their respective diagnoses, and a unanimous decision is not required[1[]](https://paperpile.com/c/8OOhuc/98Ena). For the first criterion, a risk matrix was developed to state the intensity of asbestos of the most common occupations per decade, for the period of 1945-1995. More specifically, the years of work are multiplied by the corresponding intensity factor for the patient's occupations during that time, leading to an overall grade of the intensity of total asbestos exposure, which can be converted to fiber years. This value has to be higher than five fiber years to meet the criterion of sufficient history of occupational asbestos exposure. The second criterion of lung parenchyma fibrosis is evaluated through visual radiological inspection, where an experienced reader estimates the 3D volume of fibrosis, from the 2D slices of the CT scan. The fibrosis has to cover at least 5% of the pleural surface. The third criterion is lung function loss, which is estimated on a 5-point scale based on the criteria by the American Medical Association (AMA) and "Guides to the evolution of permanent impairment," 6th edition 2008 [2]. These guidelines describe the three most indicative parameters of lung function loss of applicants with asbestosis: (1) forced vital capacity (FVC), (2) diffusing capacity for carbon monoxide (DLCO), and (3) the maximal oxygen consumption (VO2 max). FVC is the total amount of air the patient can exhale by force after a full inhalation in liters. The DLCO describes the ability of carbon monoxide (as a substitute for oxygen) to transfer into the blood in ml/min/mmHg. VO2 max is the maximal uptake of oxygen during incremental exercise in ml/min/kg. The lowest-scoring one determines the lung function loss category (Table 4.1). AMA class >1 is required to meet the third criterion. Besides the AMA classification and their corresponding lung function tests, the vital capacity (VC) is often given to assist the pulmonologists in their assessment of the lung function of the patient.

*Network Design & Implementation*
The 3D ResNet-18 architecture was implemented (Figure S1)[3]. It learned features from the CT scan (and corresponding anomaly heatmap) from 192 x 192 x 96 x 2 through multiple convolutions with striding operations to 3 x 6 x 6 x 512. The global average pooling layer compresses the feature maps to a vector representation. These 512 features are subsequently fed to the logistic classifier, which results in a corresponding probability of each class (e.g. asbestosis or no asbestosis). For the advanced combination, where the AI-system included the DLCO, an additional layer was implemented before the classification layer with four fully connected nodes to summarize the 512 pooled features of the CT image input. We implemented the lung function parameter value parallel to this layer and connected it to the classification layer. Each setup of the 3D ResNet network was trained using Tensorflow (v1.15.0) and Keras (v2.3.1) libraries on two NVIDIA GeForce RTX 2080Tis. The batch size was set at sixteen total, eight per GPU. Adam was used as optimizer, with an initial learning rate of 1e-3. The AI was trained for a maximum of 200 epochs, where early stopping was used to stop the training if the validation loss did not improve over 30 epochs. The best model checkpoint at the end of every epoch was performed. Data augmentation with rotation (up to 10^◦^) around the longitudinal axis, and flipping over the sagittal plane of the image was implemented at runtime during training.

*Variational Auto-Encoder - Anomaly Heatmap*
Variational autoencoders (VAE) are types of networks that learn to identify common features, or characteristics, of a reference "normal" population. When presented with "abnormal" cases (i.e. cases that fall outside of this reference population), the algorithm will not be able to correctly estimate these features, resulting in a deviation between the algorithm-measured value and the actual value, i.e. an anomaly. *Variational Auto-Encoder - Dataset*
To train a VAE to model healthy lung tissues, we collected a publicly-available CT dataset of lymphadenopathy patients [[4](https://paperpile.com/c/8OOhuc/F52Y)]. CT slices containing labeled enlarged lymph nodes were removed, since the dataset should only contain healthy CT slices. The dataset contained N=867 patients, corresponding to a total of N=205 519 CT scan slices.

*Variational Auto-Encoder - Data curation*To mitigate differences in imaging protocols, all CT density histograms were clipped between -1024 and 3072 Hounsfield Units (HU) and scaled on the interval [0, 1]. Slices were also resampled to 256 x 256 due to hardware constraints. To focus the attention of the VAE on the lungs, we performed segmentation of the lungs, and we blackened the background region. The segmentation was performed using a publicly-available deep learning segmentation network [[5](https://paperpile.com/c/8OOhuc/nTvbv)]. Lung segmentations were dilated through morphological operators with a kernel of 20 x 20 x 5 voxels to include adjacent tissue (i.e. thoracic wall) where pleural plaques are commonly found.

*Variational Auto-Encoder - Network Design*The proposed network design follows the standard architecture of the variational autoencoder[[6](https://paperpile.com/c/8OOhuc/Zslje)], where encoder, latent space, and decoder are placed in subsequent order. The encoder is composed of 6 convolutional blocks. Blocks are composed of repeated layers of convolutions, batch normalization, and the LeakyReLU activation function[6]. Downsampling is implemented through striding. The first block starts with 16 filters. Each subsequent block adds 16 filters. The decoder is composed of the mirrored architecture of the encoder, where the convolutional layer with stride 2 is replaced with a convolutional layer with a single stride and a subpixel upscaling layer7] at the end of the convolutional block. Sigmoid is used on the last layer of the reconstruction to constrain the image on the interval [0, 1]. While there has been some advancement in the architecture, most notably the usage of fully-convolutional layers in the latent space for medical image reconstruction[8], we kept fully connected nodes in the latent representation. This might seem disadvantageous to spatial representations, but through internal experiments, we observed that the fully connected architecture prevents the VAE from reconstructing anomalies with patches and features learned from healthy tissue. Values in the latent space are reshaped to a 4 x 4 x 96 format and passed forward to the decoder part. The decoder upsamples this vector through convolutional layers and subpixel upscaling to reconstruct the full-size image.


*Variational Auto-Encoder - Network Implementation*The VAE network was designed and trained using Tensorflow (v1.15.0) and Keras (v2.3.1) libraries on an NVIDIA GeForce RTX 2080Ti. N=195 519 slices were assigned to the training set and N=10 000 to the validation set for monitoring the training process. The batch size was set to 48. Adam was used as optimizer, with an initial learning rate of 1.5e-3. The VAE trained for 200 epochs, where the weight of the KL term in the loss was increased by 0.05 after each epoch, reaching a maximum value of 1.0 in total. Best model checkpoint at the end of every epoch was performed. Data augmentation with rotation (up to 20) and horizontal flipping of the image was implemented during training.

Figure S1: The architecture of the implemented 3D ResNet. The left column shows the encoder, where the image is downsampled through subsequent ResNet blocks to generate a prediction. The right column shows the ResNet block architecture. The black arrows represent the connections of the blocks. The blue arrows represent the identity connections, where the output of an activation layer is added to the input of another convolutional layer.

## References

## 1. Hagmolen Of Ten Have W, Rooijackers JM, Burgers JA (2016) [Financial compensation for asbestosis patients]. Ned Tijdschr Geneeskd 160:D544

## 2. Rondinelli RD, Genovese E, Katz RT, et al (2008) AMA Guides to the Evaluation of Permanent Impairment, 6th Edition

## 3. He K, Zhang X, Ren S, Sun J (2016) Deep residual learning for image recognition. In: Proceedings of the IEEE conference on computer vision and pattern recognition. pp 770–778

## 4. Armato SG 3rd, McLennan G, Bidaut L, et al (2011) The Lung Image Database Consortium (LIDC) and Image Database Resource Initiative (IDRI): a completed reference database of lung nodules on CT scans. Med Phys 38:915–931. https://doi.org/10.1118/1.3528204

## 5. LaLonde R, Bagci U (2018) Capsules for Object Segmentation. arXiv [stat.ML]

## 6. Kingma DP, Welling M (2013) Auto-Encoding Variational Bayes. arXiv [stat.ML]

## 7. Shi W, Caballero J, Huszár F, et al (2016) Real-Time Single Image and Video Super-Resolution Using an Efficient Sub-Pixel Convolutional Neural Network. arXiv [cs.CV]

8. Wang Z, Yuan H, Ji S (2019) Spatial Variational Auto-Encoding via Matrix-Variate Normal Distributions. In: Proceedings of the 2019 SIAM International Conference on Data Mining (SDM). Society for Industrial and Applied Mathematics, pp 648–656

## 
